# 

**Published:** 1918-08-10

**Authors:** 


August 10th, 1918.] [Price Twopence

### The Hospital

The Workers' Newspaper of Administrative Medicine and Institutional Life, Administration, National Insurance and Health.

CONTENTS.

**Silver Wedding Portrait of Their Majesties** ... 411

**Academic Distinction in Medicine** ... ... ... 399

**The Great War** ... ... ... ... ... 412
X. The Awakening of the People.
Why Did America Declare War?

**The Emotional Temperament** ... ... ... 405

**The Pension Minister at Leicester** .. ... ... 408

**Hospital and Institutional News** ... ... ... 401
A Doctor Twice a Prisoner—More Medical M.P.s?—Further Presentation to a Secretary—Influenza and Hospital Visitors—Co-ordination of Medical Service in Leicester—The Unpopularity of Factory Sanatoria—U.S.A. Army School of Nursing—First Aid by Aeroplane—The Mechanical Hand—Catering for Soldiers—The *Springbok Magazine*—A Call for Action by the Pharmaceutical Society—Important Gift to Stockton Hospital—A Medical Mission in Difficulties—Women and Health Appointments—Volunteer Nurses in Infirmaries—The Appointment of Ward Assistants—Vaccination Officers' Fees or Salary—Gravesend Hospital Fête—Presentation to Rev. A. Lombardini—Misapprehension of Southwark Guardians—This Week's Drug Market—To Our Readers.

CONTENTS.

**Some Gloomy Forebodings of a Medical Practitioner** .. ... ... ... ... ... ... 409

**Leicester Health Advisory Committee** ... ... 400

**The Reform of the Poor-Law** .. ... ... ... 407
Infirmary Medical Superintendents' Recommendations.

**The Matrons', Sisters', and Women's Departments** 410
The Fate of the Agitator.
Some Experiences of Training School Life: III. Are the Hardening Influences Remedial?
Round the Hospitals.
Queen Alexandra Dedicates Nursing Service Banner—The London Hospital Nurses—The 19th and 20th Centuries—Congratulations for Miss Carrie Hall—Valiant Deeds by Nurses—"Not a Gorgon Sister."

**The Woman Worker's World** ... ... ... 419

**Some Problems of To-day** ... ... ... ... 420

Place aux Enfants.

**Matrons' and Sisters' Appointments** ... ... ... 421

**The Public Health** ... ... ... ... ... 422

**The Institutional Worker** ... ... ... ... 1
III. The Steward's Department of a Large Hospital.

